# New Insights into the Structure-Function Relationship of the Endosomal-Type Na^+^, K^+^/H^+^ Antiporter NHX6 from Mulberry (*Morus notabilis*)

**DOI:** 10.3390/ijms21020428

**Published:** 2020-01-09

**Authors:** Boning Cao, Zhongqiang Xia, Changying Liu, Wei Fan, Shuai Zhang, Qiao Liu, Zhonghuai Xiang, Aichun Zhao

**Affiliations:** State Key Laboratory of Silkworm Genome Biology, Key Laboratory for Sericulture Functional Genomics and Biotechnology of Agricultural Ministry, Southwest University, Chongqing 400716, China; boningcao@hotmail.com (B.C.); zhongqx1279@163.com (Z.X.); lcyswu@163.com (C.L.); fanwei2034@163.com (W.F.); zhangshuai2297@163.com (S.Z.); lqiao317@163.com (Q.L.); xbxzh@swu.edu.cn (Z.X.)

**Keywords:** mulberry, Na^+^, K^+^/H^+^ antiporters, salt-stress, homology modeling, ion transport

## Abstract

The endosomal-type Na^+^, K^+^/H^+^ antiporters (NHXs) play important roles in K^+^, vesicle pH homeostasis, and protein trafficking in plant. However, the structure governing ion transport mechanism and the key residues related to the structure–function of the endosomal-type NHXs remain unclear. Here, the structure-function relationship of the only endosomal-type NHX from mulberry, MnNHX6, was investigated by homology modeling, mutagenesis, and localization analyses in yeast. The ectopic expression of MnNHX6 in arabidopsis and Nhx1 mutant yeast can enhance their salt tolerance. MnNHX6’s three-dimensional structure, established by homology modeling, was supported by empirical, phylogenetic, and experimental data. Structure analysis showed that MnNHX6 contains unusual 13 transmembrane helices, but the structural core formed by TM5-TM12 assembly is conserved. Localization analysis showed that MnNHX6 has the same endosomal localization as yeast Nhx1/VPS44, and Arg402 is important for protein stability of MnNHX6. Mutagenesis analysis demonstrated MnNHX6 contains a conserved cation binding mechanism and a similar charge-compensated pattern as NHE1, but shares a different role in ion selectivity than the vacuolar-type NHXs. These results improve our understanding of the role played by the structure–function related key residues of the plant endosomal-type NHXs, and provide a basis for the ion transport mechanism study of endosomal-type NHXs.

## 1. Introduction

Sodium (Na^+^) and potassium (K^+^), which are the sixth and seventh most abundant elements on earth, play essential roles in all plants. Inside plant cells, potassium is essential for the activities of many enzymes and is important in the maintenance of electrostatic balance [1]. Salt-stress, an important abiotic stress, has become a major environmental factor limiting the productivity of crop plants [2]. To adapt to salinity, a series of physiological and biochemical mechanisms occur in plants, including excluding Na^+^ from cells, sequestering Na^+^ into vacuolar compartments, and increasing K^+^ absorption [3]. Therefore, understanding plants’ salt-tolerance mechanisms is important for increasing crop production.

In plants, Na^+^, K^+^/H^+^ antiporter (NHX)-type exchangers play important roles in salt tolerance, as well as the ion and pH homeostases of intracellular compartments, because they are the critical regulators of vesicular trafficking and cell volume. They exist in almost all species groups, including fungi, bacteria, animals, and plants [3]. NHX-type exchangers use the H^+^ gradient to couple the exchange of K^+^ or Na^+^ to their gradient, and the functional diversity of the different transporter types is dependent on their cellular localization [4]. In plants, NHXs belong to the monovalent cation/H^+^ transporters (CPA1), which are part of the large CPA family [5]. Phylogenetic and sequence analyses have shown that plant NHXs can be classified into three distinct subfamilies, including the plasma membrane-localized NHXs and the intracellular-localized NHXs, which contain the vacuolar-type NHXs, and endosomal-type NHXs [3]. In *Arabidopsis thaliana*, NHX7/SOS1 and NHX8 are plasma membrane-bound transporters that are responsible for Na^+^ and lithium ion (Li^+^) tolerance, respectively, because they transport these ions from the cell to the apoplast [6]. AtNHX1–4 are vacuolar-type NHXs and are localized to the tonoplast. They play diverse roles in cell expansion, and K^+^ and pH homeostases, under normal physiological conditions. They also play a role in salt tolerance by sequestering salts into vacuolar compartments when the plant is experiencing salt-stress [6]. AtNHX5 and 6 are endosomal-type NHXs, which play important roles in protein trafficking and K^+^ and vesicle pH homeostases depending on their cellular localization to the Golgi bodies, trans-Golgi network (TGN), and prevacuolar compartment (PVC), and are crucial for growth and development [7].

The topology of NHX antiporters usually contains 12 transmembrane domains, such as AtNHX1 (determined by experiment) [8], NHE1 (determined by homology modeling and experiment) [9,10], EcNhaA (determined by crystal structure) [11], NHA2, NHE9, and PeNHX3 (determined by homology modeling) [12,13,14]. However, MjNhaP1 (from *Methanocaldococcus jannaschii*) and PaNhaP (from *Pyrococcus abyssi*) has 13 transmembrane domains (determined by crystal structure) [15,16]. The putative topology of AtNHX5 and 6 are also composed of 13 transmembrane domains, which were predicted by consensus prediction of the α-helical transmembrane regions [17]. In addition, the AtNHX1 (vacuolar-type NHX) showed equal K^+^ and Na^+^ transport rates, and mixing Na^+^ and K^+^ revealed an additive effect. However, unlike the AtNHX1 activity, LeNHX2 (endosomal-type NHX) showed a strong preference for K^+^, and the K^+^/H^+^ activity could actually be blocked by a low Na^+^ concentration. It also appeared to be insensitive to amiloride or its derivatives [18]. This suggested that the ion-transport mechanism of plant endosomal-type NHXs might differ from that of the vacuolar-type NHXs, NHE, and EcNhaA. However, to date, little is known regarding the structure governing the ion-transport mechanism of plant endosomal-type NHXs. The endosomal-type NHXs play important roles in K^+^ and vesicle pH homeostases by cation/H^+^ binding and translocation. Therefore, the study on the ion-transport mechanism of plant endosomal-type NHXs will improve our understanding of the role played in plant growth and development.

Mulberry (*Morus notabilis*) is an ecologically and economically important perennial tree. Its leaves are the main source of food for silkworms, and its fruits are very popular and nutritious [19]. In addition, mulberry can adapt well to adverse abiotic stresses, including high salinity [20]. In our previous study, we identified and cloned mulberry *NHX* gene family members (MnNHXs), and a preliminarily analysis of their functions in response to abiotic stresses was performed [21]. Here, the halotolerance levels of the MnNHXs were analyzed through heterologous expression in yeast. The results suggested that, compared with vacuolar-type MnNHXs, the endosomal-type MnNHX6 greatly enhanced the tolerance to salt-stress. The ectopic expression of MnNHX6 also increased the tolerance to salt-stress in *Arabidopsis*. To investigate the structure governing ion-transport mechanism for MnNHX6, a three-dimensional (3D) structure of MnNHX6 was established by homology modeling, which is a useful computational approach for producing reliable structural data on membrane proteins and has been successfully used for human NHE1, NHA2, NHE9, and Populus vacuolar-type PeNHX3 [10,12,13,14]. Our MnNHX6’s 3D structure was supported by empirical, phylogenetic, and experimental data. Unlike the usual 12 transmembrane helices of NHXs, MnNHX6 contains 13 transmembrane helices, but the structural core formed by the TM5-TM12 assembly is conserved. Localization analysis showed that MnNHX6 has the same endosomal localization as yeast Nhx1/VPS44, and Arg402 is important for the protein stability of MnNHX6. According to our MnNHX6 model, the functions of the conserved residues were analyzed by mutagenesis analysis in yeast, which demonstrated MnNHX6 contains a conserved cation binding mechanism and a similar charge-compensated pattern as NHE1, but shares a different role in the ion selectivity than the vacuolar-type NHXs. Overall, our results improve our understanding of the role played by the structure–function related key residues of the plant endosomal-type NHXs, and provide a basis for the ion transport mechanism study of endosomal-type NHXs.

## 2. Results

### 2.1. Halotolerance Phenotypes of MnNHX6

**Figure 1:**
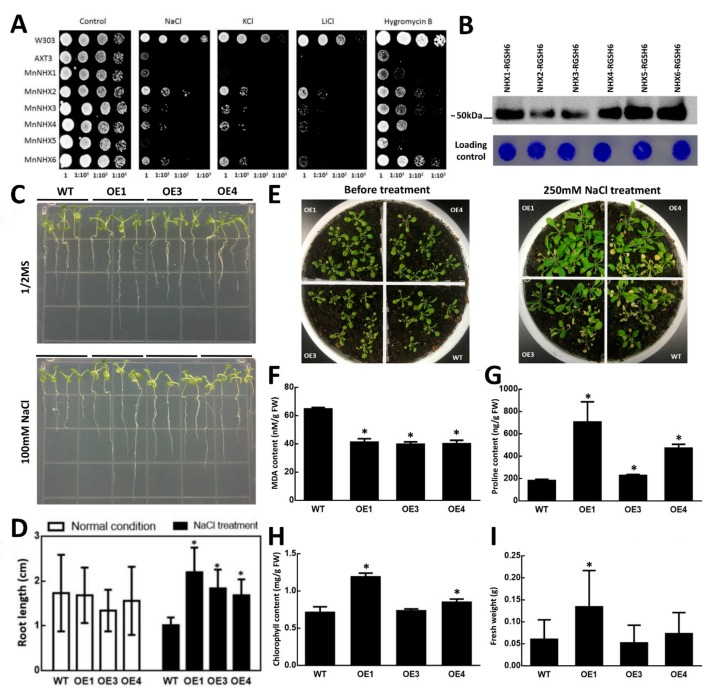
Salt-tolerance analyses of MnNHX6 in yeast and transgenic *Arabidopsis* plants. (**A**) A comparison of MnNHX family halotolerance levels in yeast. All the MnNHX family (MnNHX1–6) open-reading frames were cloned into the plasmid pYPGE15 and transformed into yeast *AXT3* cells. Wild-type (WT) yeast *W303* served as the positive control. In total, 4 μL of the 10-fold serial dilutions of these strains from saturated cultures were spotted onto AP plates supplemented with NaCl (110 mM), KCl (900 mM) or LiCl (10 mM) at pH 5.8 and YPD plates supplemented with hygromycin B (25 μg/mL). (**B**) Expression levels of the MnNHX1-6 (with RGSH6 tag) were examined. Microsomal membrane proteins (35 μg) were separated electrophoretically and were subjected to Western blot using anti-His antibody; the loading control was done by protein staining with Coomassie Brilliant Blue. (**C**) The root growth of MnNHX6 transgenic *Arabidopsis* plants (The overexpression MnNHX6 transgenic lines 1, 3, and 4 were designated as OE1, OE3, and OE4, respectively) under normal and NaCl-stress conditions. (**D**) Statistical analysis of root lengths of MnNHX6 transgenic and WT (wild-type) plants. Data are means ± SDs (*n* = 9), * *p* < 0.05. (**E**) The growth of MnNHX6 transgenic *Arabidopsis* and WT plants under NaCl-stress conditions. (**F**–**I**) The proline (**F**), malondialdehyde (MDA) (**G**), chlorophyll (**H**) contents and fresh weight (**I**) in MnNHX6 transgenic *Arabidopsis* and WT plants under NaCl-stress conditions. Data are means ± SDs (*n* = 6), * *p* < 0.05.

In our previous study, we identified all the members of the MnNHX gene family (MnNHX1–6). The phylogenetic and domain analyses suggested that MnNHX1–5 were vacuolar-type NHXs, like AtNHX1–4. However, MnNHX6 was an endosomal-type NHX, like AtNHX5 and 6 [21]. To better understand the function of MnNHXs, drop tests were used to compare the halotolerance levels of the MnNHX family members (MnNHX1–6). All of the MnNHX family open-reading frames (ORFs) were cloned into the plasmid pYPGE15 and transformed into *Saccharomyces cerevisiae AXT3* cells. The *AXT3* strain lacks the main Na^+^ transporters (ENA1–4, NHA1, and NHX1) from the wild-type yeast *W303*, resulting in sensitivities to salt, high K^+^ levels, and hygromycin B [22]. The different halotolerance levels of MnNHX family members (MnNHX1–6) are shown in Figure 1A. All the members can increase the *AXT3* strain’s tolerance to hygromycin B; however, MnNHX1 and MnNHX5 led to very subtle increases in the tolerance to hygromycin B, and they did not increase the tolerance to NaCl, KCl, or LiCl in comparison with the *AXT3* strain. MnNHX3 and MnNHX4 resulted in mild increases in the tolerance to all salt-stresses and hygromycin B. MnNHX2 and MnNHX6 led to the greatest increases in the tolerance to LiCl and hygromycin B, respectively, and shared the same tolerance capabilities as NaCl and KCl (Figure 1A). The expression level of MnNHX1–6 was examined by Western blot, and MnNHX2 and 3 showed a lower expression level compared to other MnNHXs (Figure 1B). It suggested that the expression level does not much affect the function of MnNHX2 and 3. All in all, endosomal-type MnNHX6 greatly enhanced the tolerance to salt-stress, and to hygromycin B in particular, while considering the structure governing ion-transport mechanism for plant endosomal-type NHXs remains unclear, relative to vacuolar-type MnNHXs, MnNHX6 was selected over MnNHX2 as the focus for further study.

To investigate MnNHX6’s functions in plants, the recombinant plasmid PLGNL-CaMV35S::MnNHX6 (Figure S1A) was transformed into *Arabidopsis*, and transgenic plants were produced. These transgenic lines were selected on selective medium containing kanamycin. The transgenic plants were analyzed using PCR, with genomic DNA as the template, and β-glucuronidase (GUS) staining (Figure S1B,D). The expression levels of the MnNHX6 gene in the control wild-type plants and the transgenic lines were examined using reverse transcription quantitative PCR (qRT-PCR) analyses (Figure S1C). The overexpression levels of MnNHX6 in transgenic lines 1, 3, and 4 (designated as OE1, OE3, and OE4, respectively) were greater than in the wild-type (Figure S1C). Thus, they were chosen for further analysis.

The seven-day-old seedlings of the wild-type (WT), OE1, OE, and OE4 lines were transferred to plates for another 10 d. The four lines showed similar fresh weights and root lengths under normal conditions, but the root length of the WT was seriously limited when exposed to NaCl stress (Figure 1C,D). The three-week-old lines of the WT, OE1, OE3, and OE4 were treated with 250 mM NaCl solution for 30 d. The fresh weight of the transgenic and the WT plants showed that the OE1 transgenic lines significantly increased growth compared with the WT and other transgenic plants (Figure 1I). Some important physiological indices were measured to investigate the physiological differences between transgenic lines and the WT. The leaves of the WT and OE3 transgenic lines showed more etiolated than other transgenic plants (Figure 1E), the chlorophyll content of the WT and OE3 were also lower than OE1 and OE4 (Figure 1H), and similar results were shown in the chlorophyll a (Chla) and chlorophyll b (Chlb) contents (Figure S2). The OE1, OE3, and OE4 transgenic lines showed significantly higher levels of proline compared to the WT, but lower levels of malondialdehyde (MDA) under salt-stress conditions (Figure 1F,G). The phenotyping and physiological characterization suggested that the overexpression of MnNHX6 significantly enhanced salt-stress tolerance in *Arabidopsis*.

### 2.2. The Predicted Topology of MnNHX6 Contains 13 Transmembrane Segments

**Figure 2:**
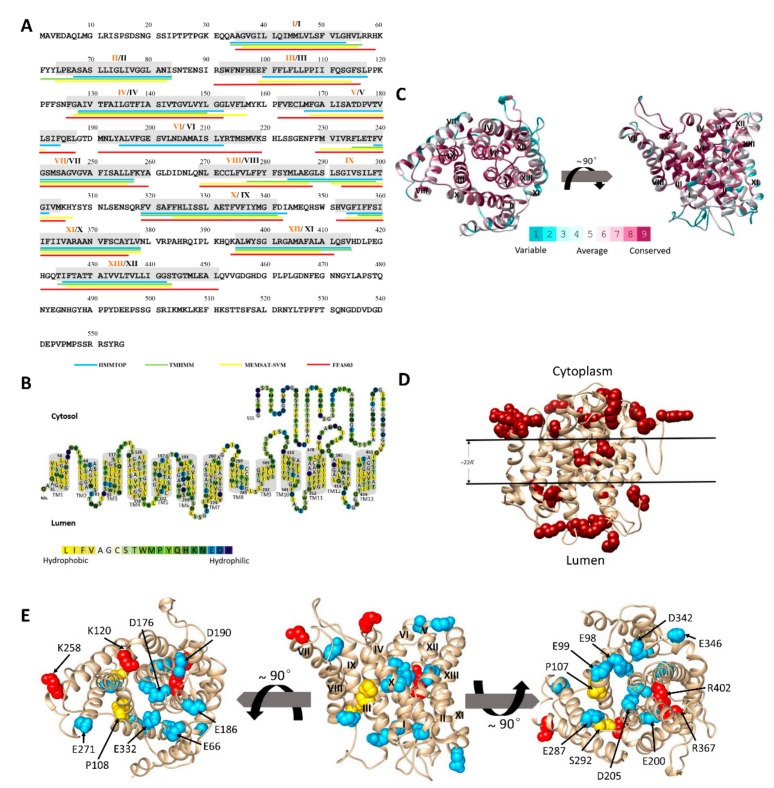
The membrane topology and three-dimensional (3D) model of MnNHX6. (**A**) The transmembrane (TM) segments and topology of the MnNHX6 sequence. The TM helices were predicted by different methods, which are underlined with different colors: HMMTOP with blue, TMHMM with green, MEMSAT-SVM with yellow, and FFAS03 with red. The final TM helix assignments are highlighted in gray, and the numbers of final TM helices are displayed in orange Roman numerals. The other TM helix predications of the endosomal-type NHXs are displayed in black Roman numerals based on the amino acid sequence alignment of AtNHX1. The differences between the two boundaries of endosomal-type NHXs are discussed in the main text. (**B**) The suggested TM topology of MnNHX6. Residues are colored according the hydrophobicity scale of Kessel and Ben-Tal [27]. Overall, the TM helices of MnNHX6 are hydrophobic, but they do feature polar and titratable residues. (**C**) The MnNHX6 model structure was supported by evolutionary conservation. Top and side views of a model structure of the membrane domain of MnNHX6 based on the structure of MjNhaP1 and colored according to the degree of ConSurf conservation. (**D**) The MnNHX6 model follows the ‘positive-inside’ rule. The lysine and arginine residues in the MnNHX6 model are shown as dark red spheres. The approximate boundaries of the hydrocarbon regions of the membrane are shown in black. (**E**) Experimental validation of the MnNHX6 model using structure-guided mutations. The site-directed mutagenesis of MnNHX6 is shown in Table S1. The distribution of these residues is shown in the MnNHX6 model. The conserved structure-related residues, and the negatively and positively charged residues, in the MnNHX6 model are shown as yellow, blue, and red spheres, respectively. Left side; a top view from the cytoplasmic side of the membrane. Middle; a side view parallel to the membrane with the cytoplasmic side facing upward and the TM segments numbered. Right side; a view from the luminal side.

Plant endosomal-type NHXs belong to the large CPA family of monovalent cation/H^+^ transporters (CPA1), but only three NHX antiporters, the Na^+^/H^+^ antiporter of *Escherichia coli* (EcNhaA), *Pyrococcus abyssi* (PaNhaP), and *Methanocaldococcus jannaschii* (MjNhaP1) presently have known crystal structures. We note that the three crystal structures suggested two different topological models, 12 (EcNhaA) or 13 transmembrane segments (PaNhaP and MjNhaP1), both of these models are biologically functional [11,15,16]. The sequence similarity level among the three NHX antiporters and MnNHX6 is less than 10%, which means that standard methods cannot be used to align their sequences. The membrane topology of MnNHX6 was predicted using FFAS03 servers, which calculated the pairwise alignments between target and template sequences. The topological models of the NHA, NHE, and NHX structures were constructed using this approach, and the EcNhaA structure was used as a template [10,12,13,14]. In this study, we identified MjNhaP1 from the Pfam database as the closest homolog to MnNHX6 using the FFAS03 server [23]. We assigned the boundaries of 13 transmembrane (TM) segments of MnNHX6 based on the corresponding segments in the crystal structure of MjNhaP1 [15] (Figure 2A). In addition, we used the HMMTOP [24], TMHMM [25], and MEMSAT-SVM [26] servers to predict the TM helices. The results were used to assign the final predicted membrane topological model (highlighted in gray in Figure 2A and illustrated in Figure 2B).

### 2.3. The 3D Model of MnNHX6 Shares a Typical ‘*Funnel*’ Fold

The MnNHX6 helix assignment was refined using FFAS03, and the predictions were made by other programs. Then, a 3D model of MnNHX6 was constructed based on this assignment and the MjNhaP1 template by using the homology modeling program SwissPdb viewer (http://us.expasy.org/spdbv/) (Figure 2C). There are obvious differences in the membrane topology between MnNHX6 and other Na^+^/H^+^ exchangers, such as NHE1, NHE9, NHA2, EcNhaA, and vacuolar-type NHXs in plants. MnNHX6 has 13 TM segments, while the other Na^+^/H^+^ exchangers contain 12 TM segments [8,9,10,11,12,13,14]. The 3D model of MnNHX6 revealed that a typical “funnel” structure, which is associated with the core of an alternating-access mechanism for Na^+^/H^+^ exchange in NHE1 and EcNhaA, was assembled by TM5–12 [10,11]. However, the typical “funnel” structures are assembled by TM4–11 in NHE1, NHE9, NHA2, EcNhaA, and vacuolar-type NHXs in plants [10,11,12,13,14]. In the MnNHX6 model, the TM5 and TM12 segments unwound to form extended peptides in the center of the helix and crossed each other in the middle of the membrane. The TM5–12 assembly was embedded between TM3, TM5, TM6, TM10, and TM12, and the external funnels were shaped by TM2, TM4, TM8, TM9, and TM13 (Figure 2C). All in all, our MnNHX6 model shares a typical “funnel” fold as a structural basis for the alternating-access mechanism.

### 2.4. Evolutionary Conservation Supports the MnNHX6 Model Structure

The evolutionary conservation score of the MnNHX6 model was projected by the ConSurf server (http://consurf.tau.ac.il/). Consistent with the reliability of the model, we showed that the model structure is compatible with the conservation pattern in that, overall, the concentrations of evolutionarily conserved residues are within the core regions of the transporter, while the variable residues face the lipids or are located in TM1, TM7, TM11, and extramembrane regions (Figure 2C). Our analysis revealed that, even though the membrane topology of MnNHX6 was different than other Na^+^/H^+^ exchangers, the TM5–12 assembly in the MnNHX6 model was conserved along with the TM4–11 assembly of EcNhaA, NHE1, and NHA2.

### 2.5. The MnNHX6 Model Structure Is Consistent with Hydrophobic Characteristics and the ‘Positive-Inside’ Rule

Structures of membrane proteins display distinct common features, especially in regard to the distribution of positively charged and hydrophobic residues. We evaluated the compatibility of the MnNHX6 model with these generic characteristics. As shown in Figure 2B, the MnNHX6 model possessed typical physicochemical properties of transporter proteins, with the most polar residues being clustered in the core or on extramembrane loops, while the hydrophobic residues faced the membrane. The “positive-inside” rule, which is an empirical observation, showed that the vast majority of TM proteins are such that amino acid positions at the intracellular regions are enriched in positively charged residues (lysine and arginine) compared with the extracellular regions, which can be used to evaluate the topology of a membrane protein [28,29]. This distribution has been readily demonstrated in EcNhaA, NHE1, and NHA2 [10,11,12]. An analysis of the MnNHX6 3D model revealed 18 arginine/lysine residues on the cytoplasmic side of the membrane and 12 on the luminal side (Figure 2D), which indicates that the MnNHX6 model follows the “positive-inside” rule.

### 2.6. Experimental Validation of the MnNHX6 Model by Structure-Guided Mutants 

To obtain structural and functional insights on MnNHX6, we undertook the site-directed mutagenesis of all the highly conserved charged residues in the model of MnNHX6, including the conserved negatively charged residues Glu66, Glu98, Glu99, Asp176, Glu186, Asp190, Glu200, Asp205, Glu271, Glu287, Glu332, Asp342, and Glu346, the conserved positively charged residues Lys120, Lys258, Arg367, and Arg402, and the conserved structure-related residues Pro107, Pro108, and Ser292 (Table S1). The distributions of these residues included the conservative core or the nonconservative extramembrane loops (Figure 2E). The aspartic and glutamic acids were substituted with asparagine and glutamine, respectively, and the positively charged residues were substituted with cysteine. Here, to study the correlations between function and protein stability of these mutants, the mutagenesis was performed using the PCR method with MnNHX6-EGFP (EGFP fused to the C-terminus of MnNHX6) used as the template. The EGFP fusion proteins did not change the MnNHX6 function (Figure S3).

**Figure 3:**
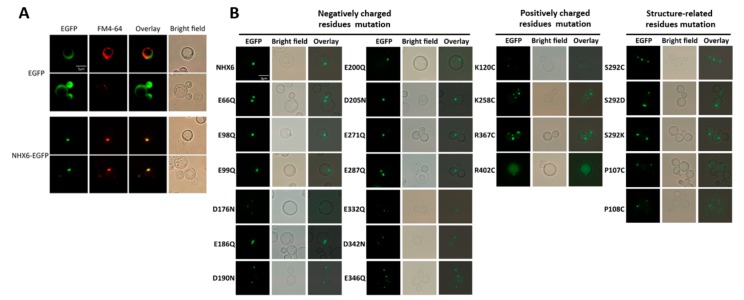
Fluorescence microscopy of MnNHX6-EGFP and the site-directed mutagenesis of MnNHX6-EGFP in yeast. (**A**) Localizations of EGFP and MnNHX6-EGFP were observed in live yeast cells under fluorescence microscopy. Vacuoles (top panel) and prevacuolar compartment (PVC) (bottom panel) of the cells expressing EGFP were stained with styryl dye *N*-(3-triethylammoniumpropyl)-4-(6-(4-diethylamino)-phenyl (FM 4-64). The cells expressing MnNHX6-EGFP in the PVC were stained with FM 4-64. The overlays of the fluorescence microscopy images are shown in the third column. MnNHX6-EGFP fluorescence was located in membranes of the PVC, which was the same localization as yeast ScNHX1 [29]. (**B**) The subcellular localizations of the site-directed yeast mutants of MnNHX6-EGFP were observed under fluorescence microscopy and bright field. The overlay images are shown in the third column. The fluorescence of the mutant R402C showed a partial missorting to the vacuole, while other mutants were correctly localized in the endosomes/PVC, similar to wild-type MnNHX6.

**Figure 4:**
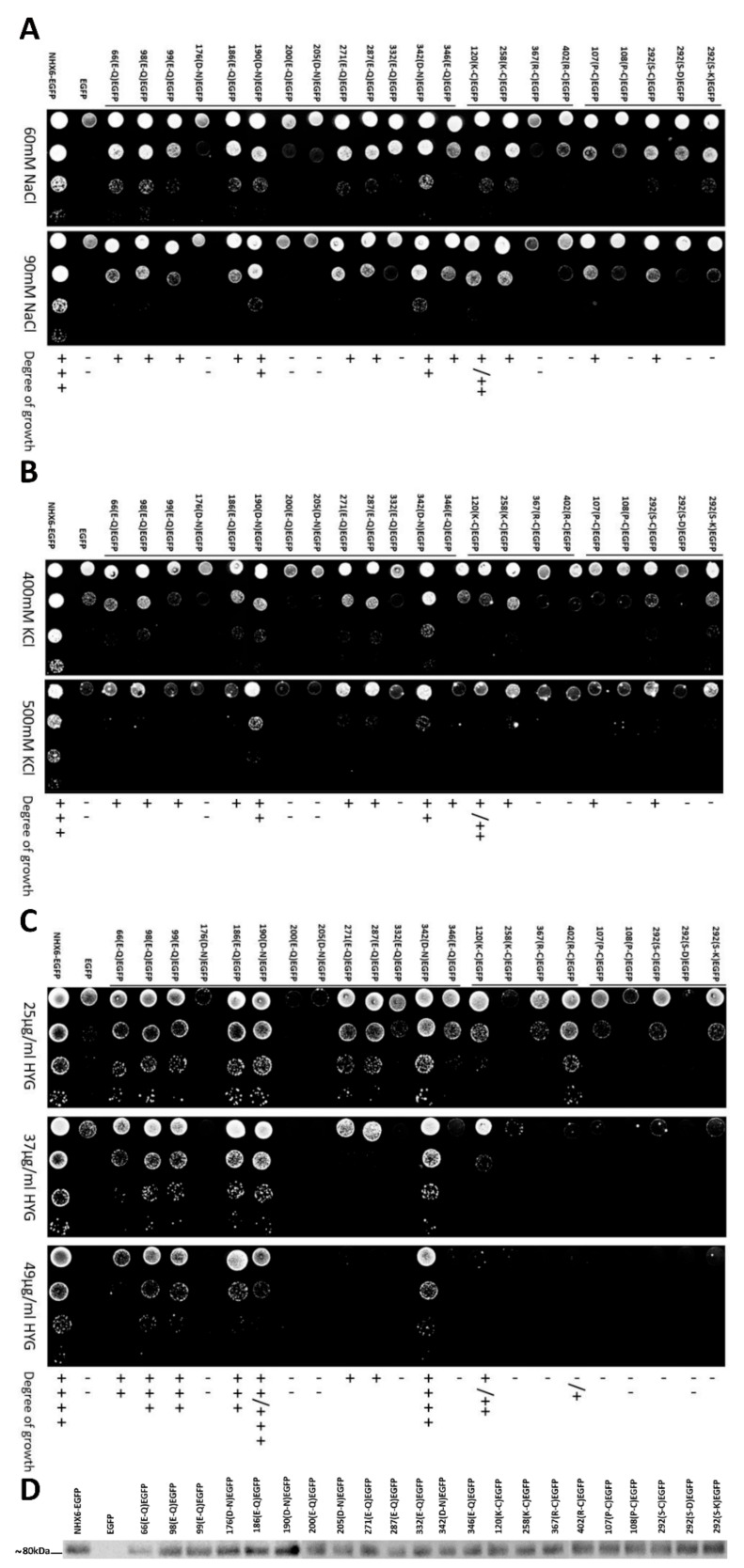
Phenotypic screening of MnNHX6 mutations in yeast. (**A**–**C**) The full-length MnNHX6 and different mutations of the MnNHX6 open-reading frame (ORF) without the stop codon were separately fused at the N-terminus of EGFP (pYPGE15-MnNHX6-EGFP). These point mutants of MnNHX6 were introduced into the yeast mutant strain *AXT3*. Empty pYPGE15-EGFP and pYPGE15-MnNHX6-EGFP were used as negative and positive controls, respectively. In total, 4 μL of 10-fold serial dilutions of these strains from saturated cultures were spotted onto AP plates supplemented with different concentrations of NaCl (60 and 90 mM) (**A**), KCl (400 and 500 mM) (**B**) at pH 5.8 and YPD plates supplemented with hygromycin B (25, 37, and 49 μg/mL) (**C**). (**D**) Expression levels of the different mutations of MnNHX6-EGFP (NHX6-EGFP). Microsomal membrane proteins (35 μg) were separated electrophoretically and subjected to Western blot using anti-GFP antibodies, pYPGE15-MnNHX6-EGFP (NHX6-EGFP) and pYPGE15-EGFP (EGFP) was used as positive and negative control, respectively.

As shown in Figure 3A (first column), the endosomal-type NHX MnNHX6-EGFP fusion protein was localized to between one and three punctate compartments, and EGFP was distributed throughout the cytoplasm of the yeast cells. Vacuoles (top panel) and PVC (bottom panel) of the cells expressing EGFP and the cells expressing MnNHX6-EGFP in the PVC were stained with FM 4-64. The fluorescence microscopy images are shown in the second column and the overlapped images are shown in the third column (Figure 3A). MnNHX6-EGFP fluorescence was located in membranes of the PVC, which was the same localization as yeast ScNHX1 [30]. In contrast, the vacuolar-type AtNHX1 was predominantly localized in the vacuole and additional punctate bodies in the yeast [22]. Functional defects caused by the endosomal/PVCs’ translation of yeast mutants, such as *nhx1*/*vps44*, led to hygromycin B sensitivity [31]. The drop test and subcellular localization of MnNHX6 in yeast (Figure 1 and Figure 4A) suggested that the endosomal-type NHXs took over the ScNHX1 endosomal/prevacuolar functions and that the roles of the endosomal-type NHXs were closer to ScNHX1 than to those of vacuolar-type NHXs. This was consistent with the evolutionary tree constructed using ScNHX1 and plant NHXs [32]. All in all, these results suggested that the endosomal-type MnNHX6 may have similar functions to the yeast NHX antiporters in Na^+^, K^+^, and endosomal trafficking.

To test the function of the mutants, all the cells transformed with the mutants were grown in medium supplemented independently with different NaCl, KCl, and hygromycin B concentrations (Figure 4). All the mutants showed functional decrease under salt-stress conditions compared with wild-type MnNHX6. Under NaCl stress, the mutants D176N, E200Q, D205N, and R367C did not show any tolerance to NaCl (Figure 4A). The mutants D190N and D342N showed subtle decreases, while the mutants E66Q, E98Q, E99Q, E186Q, E271Q, E287Q, E332Q, D346N, K120C, K258C, and R402C showed mild decreases in tolerance under NaCl-stress conditions (Figure 4A). Under KCl stress, except for the mutants D190N and D342N, which showed mild decreases in tolerance to KCl, other mutants showed significantly reduced activities compared with wild-type MnNHX6 (Figure 4B). However, the mutants D176N, E200Q, D205N, and R367C did not show any tolerance to KCl (Figure 4B). Thus, the results indicated that all the conserved charged amino acids appear to be involved in ion binding and translocation, especially in K^+^ binding and translocation. In addition, Asp176, Glu200, Asp205, and Arg367 are more essential for Na^+^/K^+^ binding and translocation functions, compared with the other conserved charged amino acids. Under hygromycin B-stress conditions, except for the mutants E98Q, E99Q, E186Q, D190N, and D342N, the other mutants abolished the function of MnNHX6, conferring growth on the hygromycin B-supplemented medium (Figure 4C). The sensitivity of *AXT3* to hygromycin B toxicity is a result of defective trafficking to the vacuole [31]. The results indicated that, except for the Glu98, Glu99, Glu186, Asp190, and Asp342, the other conserved charged amino acids are essential for trafficking to the vacuole.

The MnNHX6 model suggested that the highly conserved Pro107 and Pro108 of TM3 in MnNHX6 correspond to Pro167 and Pro168 of TM2 in NHE1, respectively, which are involved in the conformational changes of the cation-transporter [13]. The Pro107 and Pro108 of MnNHX6 were substituted with cysteine. The mutants P107C and P108C showed significantly reduced activities under salt- and hygromycin B-stress conditions, compared with wild-type MnNHX6 (Figure 4), which indicated that the functions of Pro107 and Pro108 in MnNHX6 were conserved and that the plant endosomal-type NHX shared a similar Na^+^ and K^+^/H^+^ exchange mechanism with NHE1.

The highly conserved Ser292 of TM9 in MnNHX6 is equivalent in position to Ser351 and Ser362 of TM8 in NHE1 and NHA2, respectively, and may participate in cation binding [13,14]. Ser292 of MnNHX6 was independently substituted with cysteine, aspartic acid (negatively charged residue), and lysine (positively charged residue). The mutants S292C, S292D, and S292K, respectively, showed significantly reduced activities under salt- and hygromycin B-stress conditions compared with wild-type MnNHX6 (Figure 4). Interestingly, the mutant S292D was more sensitively to salt- and hygromycin B-stress than the mutants S292C and S292K (Figure 4). We note that the mutant E287Q also showed significantly reduced activity under salt- and hygromycin B-stress, and Glu287 positioned near the Ser292 in our MnNHX6 model (Figure 2E). These results indicated that Ser292 of MnNHX6 was conserved in function. In additional, the state of charge at this conserved position might be essential for cation binding.

To ascertain whether expression level and subcellular localization affect the function of the mutants, each mutant of MnNHX6-EGFP was examined by Western blot. The mutants E66Q and D190N showed lower and higher expression levels, respectively, and the others showed similar levels of expression compared with wild-type MnNHX6-EGFP (Figure 4D). It suggested that the expression level does not much affect the function of the mutants, except the mutants E66Q and D190N. The subcellular localizations of the mutants in yeast were also observed (Figure 3B). Interestingly, the fluorescence of the mutant R402C showed a partial missorting to the vacuole, while the other mutants had correct localizations in the endosomes/PVC similar to the wild-type MnNHX6. The results indicated that the hydrophobicity of MnNHX6 was affected by mutant R402C. As shown in Figure 4, the mutant R402C showed significantly reduced activities under salt- and hygromycin B-stress conditions; these results indicated that Arg402 is important to protein function and stability of MnNHX6.

In this section, we have identified some functionally essential amino acids by functional complementation tests and subcellular localization in salt-sensitive yeast strains. The functional evaluation of the point mutants of MnNHX6 is shown in Table 1, including the functional analysis, subcellular localizations, structural characteristics, evolutionary conservation analysis, and putative roles. According to our MnNHX6 model, these essential residues lie in the center of the conserved protein core or the nonconservative extramembrane loops, which are presumably involved in charge compensated, ion translocation, or pH regulation. Reasons for the differences are discussed below.

## 3. Discussion

### 3.1. MnNHX6 Contains a Conserved Transmembrane Assembly and Shares a Similar Charge-Compensated Pattern with NHE1

In this study, we tested the ion transport activities of six NHX genes (MnNHX1–6) using a yeast growth assay. The endosomal-type MnNHX6 appeared to greatly enhance the tolerance to salt-stress, and hygromycin B-stress in particular (Figure 1A), compared with vacuolar-type MnNHXs. In previous studies, the overexpression of endosomal-type NHXs can increase salt tolerance in transgenic plants [33] and our results also suggested that the overexpression of MnNHX6 can enhance salt-stress tolerance in plants (Figure 1B,D). We aimed to study further the structure governing the ion-transport mechanism for the plant endosomal-type NHXs, but the experimental determination of the three-dimensional structure of MnNHX6 is technically difficult. Thus we used computational tools to predict the MnNHX6 structure based on the crystal structure of the MjNhaP1 antiporter.

We assigned the final boundaries of 13 TM segments of MnNHX6 based on TM helices prediction servers and homology modeling (Figure 2A,C). However, NHX antiporters, such as AtNHX1, NHE1, EcNhaA, NHA2, NHE9, and PeNHX3 usually contain 12 TM segments [8,9,10,11,12,13,14]. These NHX antiporters contain a highly conserved TM4–11 assembly, in which the TM4 and TM11 segments lie in the structural core, providing the structural basis for ion binding and translocation. In the TM4–11 assembly, these helix dipoles are charge-compensated and stabilized by the highly conserved charge residues [13,18]. In our MnNHX6 model, even though MnNHX6 contains 13 TM segments, we identified the TM5–12 assembly, in which the TM5 and TM12 segments lie in the structural core. The TM5–12 assembly is highly similar to the TM4–11 assembly (Figure 2C).

**Figure 5:**
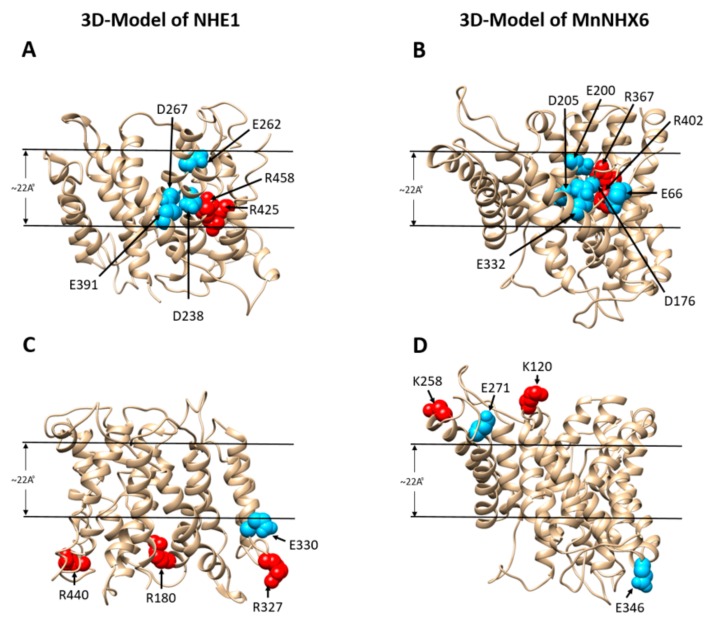
The essentially titratable residues in MnNHX6 and NHE1. Ribbon representations of a side view of the NHE1 (**A**, **C**) model [18] and our MnNHX6 (**B**, **D**) model, which are displayed with the intracellular region in the upward direction. The essentially titratable residues in the TM4–11 assembly region of NHE1 and the TM5–12 assembly region of MnNHX6 are shown in panels (**A**) and (**B**), respectively. The essentially titratable residues in the extramembrane loops of NHE1 and MnNHX6 are shown in panels (**C**,**D**), respectively. In all the panels, the negatively and positively charged residues are shown as blue and red spheres, respectively.

In addition, the MnNHX6 model suggested a charge-compensated pattern similar to that of NHE1, with Asp176 (TM5), Glu332 (TM10), Arg367 (TM11), and Arg402 (TM12) (Figure 5B) corresponding to Asp238 (TM4), Glu391 (TM9), Arg425 (TM10), and Arg458 (TM11) in NHE1, respectively (Figure 5A). A mutagenesis analysis showed that these four conserved charge residues are essential for the function of MnNHX6 (Figure 4). In addition, the D176N and R367C mutants were more sensitive than the E332Q and R402C mutants under salt- and hygromycin B-stress conditions (Figure 4). In the EcNhaA structure, the assembly forms dipoles that are only stabilized by Asp133 in TM4 and Lys300 in TM10 [18]; the corresponding residues in MnNHX6 are Asp176 and Arg367, respectively. This suggested that Asp176 and Arg367 in MnNHX6 were more conserved in evolution and function than Glu332 and Arg402.

### 3.2. Unique Features of MnNHX6′s TM5–TM12 Assembly

A unique feature of the nearby region in the TM5–12 assembly is the highly conserved Glu66 in TM2. The models of CPA1 family members (NHE1, NHE9, and PeNHX3) and bacterial EcNhaA had no conserved negatively charged residues in the equivalent position, but an equivalent (Glu215 in TM3) occurs in the CPA2 family member NHA2 [14]. The E66Q mutant significantly reduced the functional activity, but did not abolish it compared with the D176N, E332Q, R367C, and R402C mutants (Table 1), implying that this residue is important in the charge compensation of MnNHX6 but is not the main site. Overall, the evolutionary conservation and structural and mutagenesis analyses all indicate that Glu66 has a significant role in the compensation of helix dipoles in the TM5–12 assembly of MnNHX6, but whether Glu66 is involved in ion binding, pH regulation, or conformational changes remains to be elucidated.

We found the fluorescence pattern of the mutant R402C showed a partial missorting to the vacuole (Figure 3B). To compare the differences between the R402C mutant and wild-type MnNHX6, a 3D model of the R402C mutant protein was constructed in the same way. An alignment of the two 3D models was analyzed using Chimera software. There were no differences in transmembrane segments between the two 3D models (Figure S5). Thus, those findings suggested that Arg402 of MnNHX6 is vital for protein stability, possibly through the influence of charge compensation rather than protein folding.

Additionally, the K120C, K258C, E271Q, and E346Q mutants failed to rescue the salt- and hygromycin B-sensitive phenotype of the strain (Table 1). These conserved charged residues lie in the nonconservative extramembrane loops (Figure 5D), which are essential for the function of MnNHX6. Similar phenotypes were found in the model of NHE1 (Figure 5C), in which Arg180, Arg327, Glu330, and Arg440 lie in the extramembrane loops that are involved in pH regulation [10]. We speculated that the Lys120, Lys258, Glu271, and Glu346 of MnNHX6 might play similar roles pH regulation. Glu98, Glu99, and Glu186 also lie in the nonconservative extramembrane loops, and the E98Q, E99Q, and E186Q mutants failed to rescue the salt-sensitive, but not the hygromycin B sensitive, phenotype (Table 1). This indicated that the three residues might be involved in the cation transport pathway, but not through pH regulation. The roles of the three residues should be further investigated.

### 3.3. The Endosomal-Type MnNHX6 Shares Similar Transport Mechanisms with NHE1, but Differ from the Vacuolar-Type NHXs

**Figure 6:**
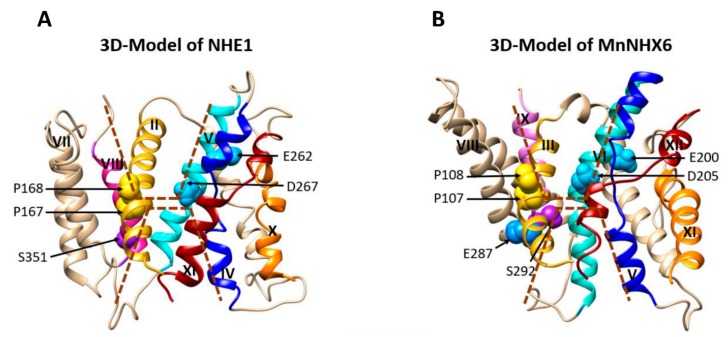
MnNHX6 and NHE1 share similar transport mechanisms. (**A**) In the NHE1 model, the cation-transport pathway is formed by two discontinuous funnels comprised of TM2, 4, 5, and 9 at the cytoplasmic side and TM2, 8, and 11 at the luminal side. Pro167 and Pro168 are located at the bend of the TM2 helix and play important structural roles for ion translocation. Glu262 (TM5) is located in proximity to the cytoplasm and might attract protons following intracellular acidification. Asp267 (TM5) and Ser351 (TM8) are located at the bottom of the cytoplasmic and luminal funnels, respectively, and serve as the main cation-binding sites [13]. (**B**) In our MnNHX6 model, two similar discontinuous funnels exist. They are formed byTM3, 5, 6, and 10 at the cytoplasmic side and TM3, 9, and 12 at the luminal side, with Pro107 (TM3), Pro108 (TM3), Glu200 (TM6), Asp205 (TM6), and Ser292 (TM9) corresponding to Pro167 (TM2), Pro168 (TM2), Glu262 (TM5), Asp267 (TM5), and Ser351 (TM8) in NHE1, respectively.

The conserved transmembrane assembly in both MnNHX6 and NHE1, as well as the similar charge-compensated patterns within them, indicated that they share similar transport mechanisms. In the NHE1 model, the cation-transport pathway is formed by two discontinuous funnels comprised of TM2, 4, 5, and 9 at the cytoplasmic side and TM2, 8, and 11 at the luminal side (Figure 6A) [10]. The experimental data of NHE1 showed that Pro167 and Pro168, which are located at the bend of the TM2 helix, play important structural roles in ion translocation. Glu262 (TM5) is located in proximity to the cytoplasm and might attract protons following intracellular acidification. Asp267 (TM5) and Ser351 (TM8) are located at the bottom of the cytoplasmic and luminal funnels, respectively, and serve as the main cation-binding sites [10]. In our MnNHX6 model, the similar two discontinuous funnels made of TM3, 5, 6, and 10 at the cytoplasmic side and TM3, 9, and 12 at the luminal side contain Pro107 (TM3), Pro108 (TM3), Glu200 (TM6), Asp205 (TM6), and Ser292 (TM9), which correspond to Pro167 (TM2), Pro168 (TM2), Glu262 (TM5), Asp267 (TM5), and Ser351 (TM8) in NHE1, respectively (Figure 6B). The mutagenesis analysis showed that these conserved MnNHX6 residues are essential for the function (Figure 4) and it suggested that MnNHX6 might share similar catalytic mechanisms with their human counterparts in NHE1.

Additionally, we note that the mutant S292D was more sensitively to salt- and hygromycin B-stress than the mutants S292C and S292K (Figure 4). However, the corresponding residue in MdNHX1 (vacuolar-type NHX) from apple (*Malus domestica Borkh.*) was Ser275 (Figure S4), and the phosphorylation of Ser275 enhanced its Na^+^/H^+^ transport activity of MdNHX1 [34]. As we know, aspartic acid is usually used to simulate the phosphorylation of serine [35]. The negative charge at this conserved position enhanced Na^+^/H^+^ transport activity in the vacuolar-type NHX (MdNHX1) but abolished the function of the endosomal-type NHX (MnNHX6). Additionally, the main difference of the topological models between AtNHX1 (vacuolar-type NHX) and MnNHX6 were in the TM8-9 segments (Figure 2A). The membrane topological model of AtNHX1was determined by protease protection assays [8], which showed that the TM8 was formed by a continuous helix (Figure S4). In the MnNHX6 model, the corresponding segments were broken into two shorter helixes; Ser292 is located between the TM8 and 9 helix. It suggested that this conserved serine of endosomal-type NHXs plays a different role in ion selectivity compared with the vacuolar-type NHXs; the ion-transport mechanism of plant endosomal-type NHXs might be different to the vacuolar-type NHXs.

In conclusion, the MnNHX6 3D structure was established by homology modeling in this study. Our MnNHX6 model was supported by empirical, phylogenetic and experimental data. The results demonstrate that MnNHX6 has unique membrane topology, charge compensation features, and ion selectivity, while sharing a conserved transmembrane assembly and similar catalytic mechanism with NHE1. This model structure may facilitate understanding of the role played by the structure-function related key residues of the plant endosomal-type NHXs, and provides a basis for further functional study of endosomal-type NHXs in growth and development as it relates to ion translocation, cation specificity and selectivity, and pH-regulation.

## 4. Materials and Methods

### 4.1. Plant Transformation and Stress-Tolerance Analysis of Transgenic Plants

The expression plasmid pLGNL carrying the MnNHX6 fragment was transformed into the GV3101 *Agrobacterium tumefaciens* strain GV3101 and used for *A. thaliana* (ecotype Columbia) transformation by the floral dip method [36]. Transgenic plant seeds were screened on 1/2 MS medium containing a final concentration of 50 mg/L kanamycin, and the grown seedlings were then confirmed as transgenic by GUS staining and genomic PCR. Additionally, the expression levels of MnNHX6 in transgenic plants were assessed by qRT-PCR analysis. A phenotypic analysis of transgenic *Arabidopsis* plants overexpressing MnNHX6 was performed in T3 plants from lines 1, 3, and 4 (OE1, OE3, and OE4, respectively). For the root growth analysis, the transgenic lines and WT *Arabidopsis* samples were grown on the normal 1/2 MS agar medium without NaCl for 7 days. Then the seven-day-old seedlings were independently incubated in 1/2 MS agar medium supplemented with 250 mM NaCl for 7 days, and the root growth was recorded. Each sample was replicated using three plates. The transgenic lines and WT *Arabidopsis* samples were grown on 1/2 MS medium until root formation and then transferred into soil in a climate chamber (25 °C, 16-h day/8-h night). After ~3 weeks, they were irrigated with water containing 250 mM NaCl for 4 weeks. Each sample was replicated using three plates. After stress treatments, fresh leaves of transgenic and WT *Arabidopsis* lines were immediately collected, and the malondialdehyde and proline contents were measured using their respective test kits (Jiancheng Bioengineering Institute, Nanjing, China) according to the manufacturer’s instructions. For chlorophyll content measuring, a 0.1g leaf sample was taken in a test tube, and 10 mL of 95% ethanol was added and kept for one day. The absorbance of solution was read using spectrophotometer at wavelengths of 645, 652, and 663 nm against solvent blank. The chlorophyll content was calculated using the formulae: Total chlorophyll (mg/g tissue) = A652 × V/(34.5 × W); chlorophyll a (mg/g tissue) = 12.7 × A663–2.69 × A645; chlorophyll b (mg/g tissue) = 22.9 × A645–4.68 × A663 [37]. Where, V = final volume of chlorophyll extract (mL) and W = fresh weight of tissue extract (g). Each sample included seven plants and was measured five times. The primers for these experiments are shown in Table S1.

### 4.2. Yeast Strain, Medium, and Plasmids

*S. cerevisiae* strains *AXT3* (Δena1-4::HIS3, Δnha1::LEU2, and Δnhx1::TRP1) and wild-type *W303* have been described previously [17]. In the drop tests, yeast cells were first cultivated in YPD (1% yeast extract, 2% peptone, and 2% glucose), and then transferred to selective AP medium (10 mM L-arginine, 8 mM phosphoric acid, 0.2 mM CaCl_2_, 2 mM MgSO_4_, 0.2 mM CaCl_2_, 2% glucose plus vitamins and trace elements, adjusted to pH 5.8 with phosphoric acid) at 30 °C until they became saturated. The saturated cultures were diluted to an OD600 of 0.5 ± 0.01, and then 4 μL of several serial (10^−1^) dilutions were spotted on AP plates supplemented with NaCl, KCl, or LiCl at pH 5.8 and YPD plates containing hygromycin B. The cation tolerances of the mulberry NHX gene family members were compared by independently cloning the ORFs for MnNHX1–6 into the yeast expression plasmid pYPGE15, and the RGSH6 tag was separately fused at the C-terminus of MnNHX1–6 for Western blotting. These constructs were introduced into the yeast mutant strain *AXT3*. The empty pYPGE15 was introduced into wild-type *W303* and *AXT3* as the positive and negative controls, respectively. Drop tests were performed as described above.

### 4.3. Preparation of Yeast Microsomal Membranes and Western Blotting

*AXT3* cells bearing appropriate pYPGE15 plasmids were grown in 5 mL SD medium and grown at 30 °C to saturation, and 3 mL of this preculture was transferred into 500 mL SD medium to saturation. This culture was then inoculated overnight into 3000 mL YPD medium. The cells were harvested, washed, and broken in 100 mM Tris-HCl (pH 7.5), 2 mM EDTA, 1 mM dithiothreitol, 20% glycerol, 1 mM phenylmethylsufonyl fluoride, and 1 mM protease inhibitor cocktail (Sigma) (Saint Louis, MO, USA) by vortexing with glass beads. The lysate was centrifuged at 30,000× *g* for 30 min, and the supernatant was centrifuged at 100,000× *g* for 90 min to obtain the microsomal fraction. Proteins (35 μg) were separated by SDS-PAGE, then subjected to Western blot using anti-His antibody and anti-GFP antibody; the loading control was done by protein staining with Coomassie Brilliant Blue.

### 4.4. Protein Structure Prediction and Evolutionary Conservation Analysis

We predicted the putative TM regions of MnNHX6 using different methods. HMMTOP [24], TMHMM [25], and MEMSAT-SVM [26] were used to predict the TM helices. HMMTOP and TMHMM are two classical programs, while MEMSAT-SVM is a more recently developed program. FFAS03 was used to identify the protein folds using profile–profile sequence alignments [23]. The FFAS03 server and the Pfam database identified the structure of MjNhaP1 as the best template for MnNHX6. The pairwise alignment between MnNHX6 and MjNhaP1 [15] enabled the construction of a 3D model of MnNHX6 using the homology modeling program SwissPdb viewer (http://us.expasy.org/spdbv/). The Consurf web server was used to calculate evolutionary conservation scores by the Bayesian method [38]. The 3D models are compatible with the evolutionary conservation analysis of NHE. The model structure was visualized using Chimera software (San Francisco, CA, USA) [39].

### 4.5. Site-Directed Mutagenesis and Microscopy

Each single point mutation of MnNHX6 was constructed using PCR with appropriate primer sets (Table S2) and confirmed by sequencing. The full-length MnNHX6 and different mutations of MnNHX6 ORFs without the stop codon were separately fused at the EcoRI site of the N-terminus of EGFP (pYPGE15-MnNHX6-EGFP). The resulting fusions were inserted between the BamHI and KpnI sites in pYPGE15. These constructs were introduced into *AXT3* cells. For the yeast expression assay, the point mutants of MnNHX6 were introduced into the yeast mutant strain *AXT3*. Empty pYPGE15-EGFP and pYPGE15-MnNHX6-EGFP were used as negative and positive controls, respectively. Drop tests were performed as described above. In the subcellular localization experiments, the cells (0.7–1.2 OD600 units/mL) of EGFP and MnNHX6-EGFP were collected by centrifugation at 2000× *g* for 1 min. Media was discarded, the cells were resuspended in YPD+FM4-64 (*N*-(3-triethylammoniumpropyl)-4-(6-(4-diethylamino)-phenyl)), and immediately placed on ice for 10 min. Cells were collected by centrifugation and resuspended in 200 μL of ice-cold YPD without FM 4-64. Cells were placed at room temperature to initiate endocytosis of the dye and observed at 10 min intervals [40]. Images were taken using an inverted fluorescence microscope (Olympus IX73, Olympus Corporation, Tokyo, Japan).

### 4.6. Statistical Analyses

The results were organized and analyzed using GraphPad Prism 6.0 (GraphPad Software, La Jolla, CA, USA). The significant differences between different samples were analyzed using SPSS Statistics 17.0 (SPSS Inc., Chicago, IL, USA) software. The means were compared using the Duncan’s test calculated at *p* < 0.05. Mean values that were significantly different from each other were indicated by asterisks.

## Figures and Tables

**Table 1:** Functional evaluation of the point mutants of MnNHX6 in yeast, including functional analysis, subcellular localizations, structural characteristics, evolutionary conservation analysis, and putative roles.

	Functional Damage			Subcellular			Conservation	Essential	Putative
Mutant	NaCl	KCl	HYG	Localization	Residue	Location	Score	Residue (Y/N)	Role
E66Q	Mild	Significant	Significant	PVC	Glu66	Core	8	Y	Charge compensated
E98Q	Mild	Significant	Subtle	PVC	Glu98	Extramembrane loop	6	Y	Unclear
E99Q	Mild	Significant	Subtle	PVC	Glu99	Extramembrane loop	6	Y	Unclear
D176N	Abolish	Abolish	Abolish	PVC	Asp176	Core	9	Y	Charge compensated
E186Q	Mild	Significant	Subtle	PVC	Glu186	Extramembrane loop	7	Y	Unclear
D190N	Subtle	Mild	Subtle	PVC	Asp190	Extramembrane loop	8	N	-
E200Q	Abolish	Abolish	Abolish	PVC	Glu200	Core	9	Y	Ion translocation
D205N	Abolish	Abolish	Abolish	PVC	Asp205	Core	9	Y	Ion translocation
E271Q	Mild	Significant	Abolish	PVC	Glu271	Extramembrane loop	9	Y	pH regulation
E287Q	Mild	Significant	Abolish	PVC	Glu287	Core	8	Y	Ion translocation
E332Q	Mild	Significant	Abolish	PVC	Glu332	Core	9	Y	Charge compensated
D342N	Subtle	Mild	Subtle	PVC	Asp342	Extramembrane loop	7	N	-
D346N	Mild	Significant	Abolish	PVC	Glu346	Extramembrane loop	6	Y	pH regulation
K120C	Mild	Significant	Abolish	PVC	Lys120	Extramembrane loop	5	Y	pH regulation
K258C	Mild	Significant	Abolish	PVC	Lys258	Extramembrane loop	9	Y	pH regulation
R367C	Abolish	Abolish	Abolish	PVC	Arg367	Core	9	Y	Charge compensated
R402C	Mild	Significant	Abolish	Partial missorting	Arg402	Core	9	Y	Charge compensated
P107C	Mild	Significant	Abolish	PVC	Pro107	Core	9	Y	Ion translocation
P108C	Mild	Significant	Abolish	PVC	Pro108	Core	9	Y	Ion translocation
S292C/D/K	Mild	Significant	Abolish	PVC	Ser292	Core	9	Y	Ion translocation

Based on the strain growth, four degrees of functional damage were characterized: subtle (subtle decrease in tolerance), mild (mild decrease in tolerance), significant (significantly reduced activity), and abolish (abolishment of activity). The Y (Yes) and N (No) represent essential residues or unessential residues in the function of MnNHX6, respectively. A line (-) indicates that the residue was nonfunctional in MnNHX6.

## Supplementary Materials

The following are available online at https://www.mdpi.com/1422-0067/21/2/428/s1. The MnNHX6 structural file (MnNHX6.pdb) used in this work; Figure S1: Confirmation of transgenic arabidopsis plants; Figure S2: The chlorophyll a (Chla) and chlorophyll b (Chlb) contents in MnNHX6 transgenic Arabidopsis and WT plants under NaCl-stress conditions; Figure S3: Growth of the strains on the YPD supplemented with or without hygromycin B; Figure S4: The alignment of the amino acid sequences of plant vacuolar-type NHX and endosomal-type NHX; Figure S5: Comparison of the transmembrane domains in 3-D models of MnNHX6 and R402C; Table S1: Site-directed mutagenesis of all the highly conserved charged residues in the model of MnNHX6; Table S2. Primers used in this work.

## Abbreviations

NHX	Na^+^, K^+^/H^+^ antiporters
TM	transmembrane
3D	three-dimensional
WT	wild-type
OE	overexpressing
MDA	malondialdehyde
qRT-PCR	reverse transcription quantitative PCR
TGN	trans-Golgi network
PVC	prevacuolar compartment
HYG	hygromycin B
EGFP	enhanced green fluorescent protein
