# Supplementary material for: New Insights into the Structure-Function Relationship of the Endosomal-Type Na+, K+/H+ Antiporter NHX6 from Mulberry (Morus notabilis)
Source: Int J Mol Sci. 2020 Jan 9;21(2):428. doi: 10.3390/ijms21020428 (PMC7014192; doi:10.3390/ijms21020428)
Supplement: Supplementary file 1 [file ijms-21-00428-s001.pdf]

Article

New Insights into the Structure-Function Relationship of the Endosomal-Type Na^+, K^+/H^+ Antiporter NHX6 from Mulberry (*Morus notabilis*)

Boning Cao, Zhongqiang Xia, Changying Liu, Wei Fan, Shuai Zhang, Qiao Liu, Zhonghuai Xiang and Aichun Zhao *

State Key Laboratory of Silkworm Genome Biology, Key Laboratory for Sericulture Functional Genomics and Biotechnology of Agricultural Ministry, Southwest University, Chongqing 400716, China; boningcao@hotmail.com (B.C.); zhongqx1279@163.com (Z.X.); lcyswu@163.com (C.L.); fanwei2034@163.com (W.F.); zhangshuai2297@163.com (S.Z.); lqiao317@163.com (Q.L.); xbxzh@swu.edu.cn (Z.X.)

* Correspondence: zhaoaichun@hotmail.com; Tel.: +86-23-6825-1803; Fax: +86-23-6825-1128

Received: 21 December 2019; Accepted: 06 January 2020; Published: date

Supplemental data

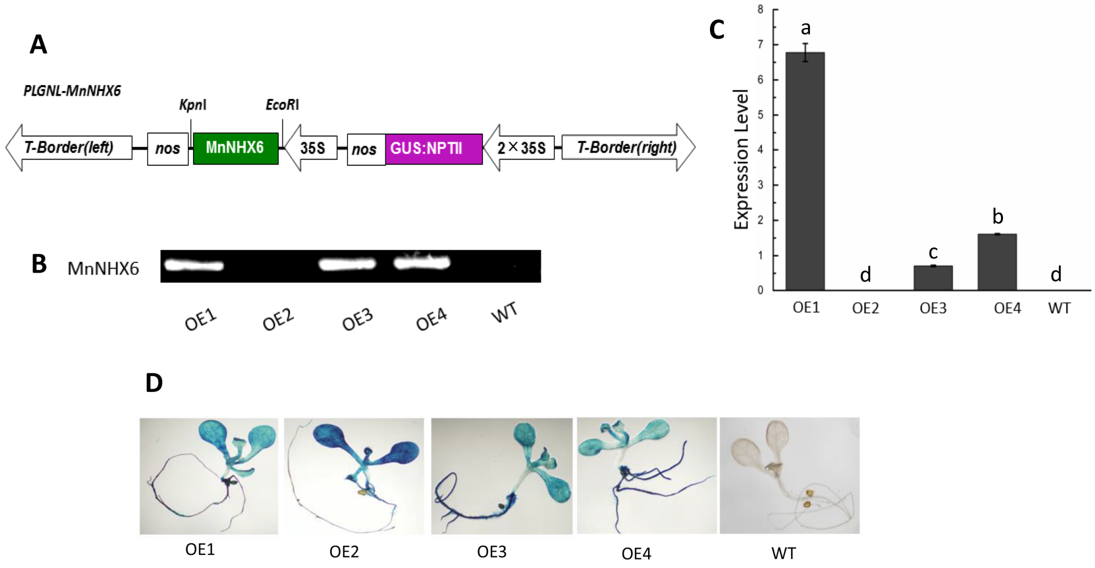

Figure S1. Confirmation of transgenic arabidopsis plants. (**A**) Diagram of the transgenic vector. (**B**) Genomic PCR analysis of transgenic lines. (**C**) Quantitative real-time PCR. Data are means ± SDs (n = 3), $p < 0.05$. Significant differences are indicated by different letters above the bar. (**D**) Histochemical GUS staining of transgenic lines.

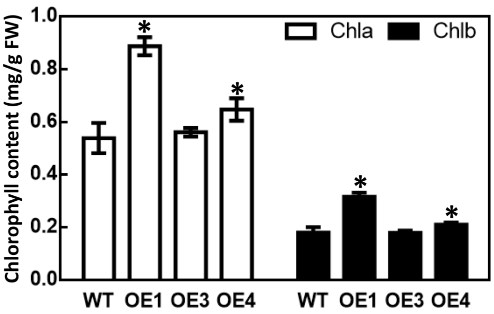

Figure S2. The chlorophyll a (Chla) and chlorophyll b (Chlb) contents in MnNHX6 transgenic Arabidopsis and WT plants under NaCl-stress conditions. Data are means ± SDs (n = 6), *P < 0.05.

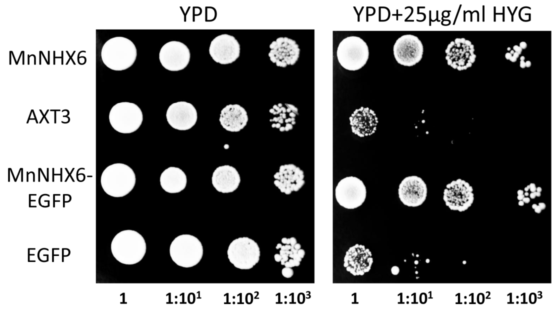

Figure S3. Growth of the strains on the YPD supplemented with or without hygromycin B. The MnNHX6 open-reading frames were cloned into the plasmid pYPGE15 and the plasmid pYPGE15-EGFP without the stop codon. These constructs were introduced into the yeast mutant strain *AXT3*. The *AXT3* (empty pYPGE15) and EGFP (pYPGE15-EGFP) were used as negative controls. 4 μL of 10-fold serial dilutions of these strains from saturated cultures were spotted onto the YPD plates with or without hygromycin B (25μg/mL).

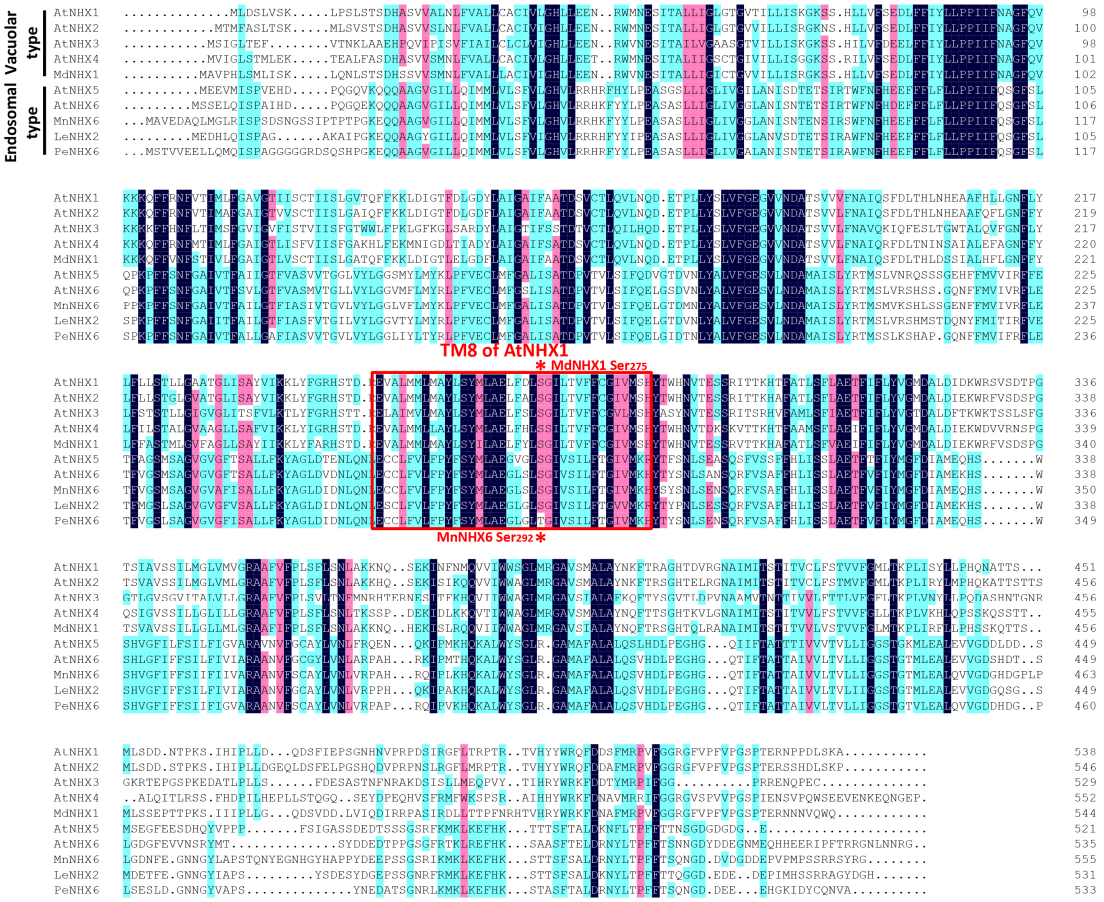

Figure S4. The alignment of the amino acid sequences of plant vacuolar-type NHX and endosomal-type NHX. The TM8 segment of AtNHX1 is marked with red box, which had been to determine by experiment [1]. The Ser275 (MdNHX1) [2] and Ser292 (MnNHX6) are marked with red asterisk.

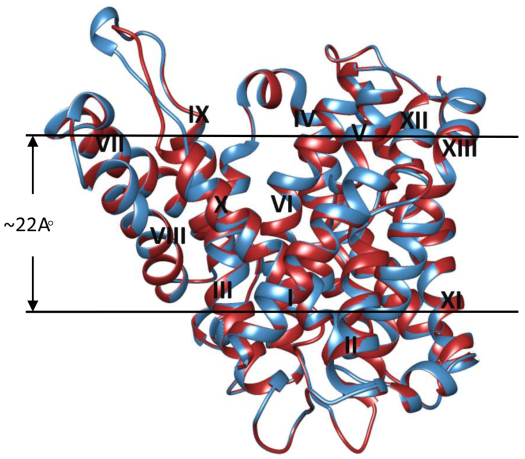

Figure S5. Comparison of the transmembrane domains in 3-D models of MnNHX6 (Wild-type, brown) and R402C (mutant, blue), the TM segments are marked with the black Roman numerals.

Table S1. Site-directed mutagenesis of all the highly conserved charged residues in the model of MnNHX6 were shown, including the conserved negatively charged residues Glu66, Glu98, Glu99, Asp176, Glu186, Asp190, Glu200, Asp205, Glu271, Glu287, Glu332, Asp342, Glu346, the conserved positively charged residues Lys120, Lys258, Arg367, Arg402, and the conserved structure-related residues Pro107, Pro107 and Ser292.

**MnNHX6**	**Location**	**Conservation score**
E66Q	TM2	8
E98Q	TM3	6
E99Q	TM3	6
D176N	TM5	9
E186Q	TM5-TM6	7
D190N	TM5-TM6	8
E200Q	TM6	9
D205N	TM6	9
E271Q	TM7-TM8	9
E287Q	TM8-TM9	8
E332Q	TM10	9
D342N	TM10-TM11	7
E346Q	TM10-TM11	6
K120C	TM3-TM4	5
K258C	TM7-TM8	9
R367C	TM11	9
R402C	TM12	9
P107C	TM3	9
P108C	TM3	9
S292C<br>S292D<br>S292K	TM8-TM9	9

Table S2. Primers used in this work.

**Primer**	**Sequence**	**Use**
MnNHX6-F1	ATGGCGGTTGAGGACG	Cloning of MnNHX6 (without the stop codon were fused the EGFP)
MnNHX6-R1	CTAACCGCGGTAACTCCTTCTAGAA	
E66Q F	TTCTATTATCTTCCACAGGCCAGTGCTTCC	
E66Q R	CTGTGGAAGATAATAGAATTTGTGACGGCG	
E98Q F	TGGTTCAATTTTCACCAGGAGTTTTTCTTC	
E98Q R	CTGGTGAAAATTGAACCATGATCTGATGCT	

E99Q F	TTCAATTTTCACGAGCAGTTTTTCTTCCTG	Point mutations of the conserved negatively charged residues of MnNHX6
E99Q R	CTGCTCGTGAAAATTGAACCATGATCTGAT	
D176N F	CTTATATCAGCGACTAATCCGGTCACTGTTTTG	
D176N R	ATTAGTCGCTGATATAAGAGCACCAAACATTAG	
E186Q F	TTGTCTATATTTCAGCAACTCGGCACGGAT	
E186Q R	TTGCTGAAATATAGACAAAACAGTGACCGG	
D190N F	CAGGAACTCGGCACGAATATGAACCTATAT	
D190N R	ATTCGTGCCGAGTTCCTGAAATATAGACAA	
E200Q F	GCCTTGGTTTTTGGGCAATCCGTCTTGAAT	
E200Q R	TTGCCCAAAAACCAAGGCATATAGGTTCAT	
D205N F	GAATCCGTCTTGAATAATGCTATGGCAATT	
D205N R	ATTATTCAAGACGGATTCCCCAAAAACCAA	
E271Q F	AACCTGCAGAACTTGCAGTGTTGTCTATTT	
E271Q R	CTGCAAGTTCTGCAGGTTGTCAATGTCTAA	
E287Q F	TCGTACATGCTTGCACAAGGTCTTAGCCTC	
E287Q R	TTGTGCAAGCATGTACGAGAAATATGGAAA	
E332Q F	ATATCATCACTCGCACAGACATTTGTTTTT	
E332Q R	CTGTGCGAGTGATGATATCAAATGAAAAAA	
D342N F	ATATACATGGGCTTCAATATCGCCATGGAA	
D342N R	ATTGAAGCCCATGTATATAAAAACAAATGT	
E346Q F	TTCGATATCGCCATGCAACAGCATAGCTGG	
E346Q R	TTGCATGGCGATATCGAAGCCCATGTATAT	
K120C F	TTCAGTCTTCCACCTTGTCCTTTCTTCTCA	Point mutations of the conserved positively charged residues of MnNHX6
K120C R	ACAAGGTGGAAGACTGAATCCTGACTGAAA	
K258C F	TCTGCTTTGCTTTTTTGCTATGCAGGGTTA	
K258C R	GCAAAAAAGCAAAGCAGAAATAAATGCAAC	
R367C F	TTTATCATAGTTGCATGTGCAGCAAATGTC	
R367C R	ACATGCAACTATGATAAATATAATTGAGAA	
R402C F	TGGTACAGTGGACTCTGTGGGGCTATGGCT	
R402C R	ACAGAGTCCACTGTACCAAAGGGCTTTCTG	
P107C-F	TTCCTGTTTCTCTTATGTCCAATCATATTT	Point mutations of the conserved structure-related residues of MnNHX6
P107C-R	ACATAAGAGAAACAGGAAGAAAAACTCCTC	
P108C-F	CTGTTTCTCTTACCTTGTATCATATTTCAG	
P108C-R	ACAAGGTAAGAGAAACAGGAAGAAAAACTC	
S292C-F	GAAGGTCTTAGCCTCTGTGGTATTGTGTCA	
S292C-R	ACAGAGGCTAAGACCTTCTGCAAGCATGTA	
S292D-F	GAAGGTCTTAGCCTCGATGGTATTGTGTCA	
S292D-R	ATCGAGGCTAAGACCTTCTGCAAGCATGTA	
S292K-F	GAAGGTCTTAGCCTCAAAGGTATTGTGTCA	
S292K-R	TTTGAGGCTAAGACCTTCTGCAAGCATGTA	

References

1. Yamaguchi, T.; Apse, M.P.; Shi, H.; Blumwald, E. Topological analysis of a plant vacuolar Na^+/H^+ antiporter reveals a luminal C terminus that regulates antiporter cation selectivity. *P. Natl. Acad. Sci. USA* **2003**, 100, 12510-12515.
2. Sun, M.H.; Ma, Q.J.; Hu, D.G.; Zhu, X. P., You, C.X.; Shu, H.R. The glucose sensor mdhxk1 phosphorylates a tonoplast Na^{+}/H^+ exchanger to improve salt tolerance. *Plant Physiol*. **2018**, 176, pp.01472.
